# Bioaccessibility of Phenolic Acids and Flavonoids from Buckwheat Biscuits Prepared from Flours Fermented by Lactic Acid Bacteria

**DOI:** 10.3390/molecules27196628

**Published:** 2022-10-06

**Authors:** Henryk Zieliński, Wiesław Wiczkowski, Joanna Topolska, Mariusz Konrad Piskuła, Małgorzata Wronkowska

**Affiliations:** Division of Food Sciences, Department of Chemistry and Biodynamics of Food, Institute of Animal Reproduction and Food Research, Polish Academy of Sciences, Tuwima 10, 10-748 Olsztyn, Poland

**Keywords:** fermented buckwheat flours, biscuits, digestion, phenolic acids, flavonoids, bioaccessibility index

## Abstract

The literature reports that the consumption of common buckwheat (*Fagopyrum esculentum* Moench), exactly the polyphenols it contains, is associated with a wide spectrum of health benefits. Therefore, the determination of the bioaccessibility of phenolic acids and flavonoids from buckwheat biscuits formulated from liquid-state fermented flours (BB_F_) by selected lactic acid bacteria (LAB) after gastrointestinal digestion was addressed in this study. Bioaccessibility could be defined as the fraction of a compound that is released from the food matrix in the gastrointestinal lumen and used for intestinal absorption. The bioaccessibility of eight phenolic acids (protocatechuic, vanillic, syringic ferulic, caffeic, sinapic, *p*-coumaric, and *t*-cinnamic) and six flavonoids (epicatechin, vitexin, orientin, apigenin, kaempferol, and luteolin) were provided for BB_F_ and BB_C_ (buckwheat biscuits prepared from fermented and unfermented flours, respectively). The bioaccessibility indexes (BI) indicated the high bioaccessibility of phenolic acids and improved bioaccessibility of flavonoids from BB_F_. Moreover, the data provide evidence for the suitability of selected LAB strains to be used as natural sour agents for further bakery product development rich in phenolic acids and flavonoids with LAB-dependent bioaccessibility.

## 1. Introduction

Common buckwheat is known as a gluten-free pseudocereal utilized worldwide, while other species are used as a traditional food in some regions such as south of China, Bhutan, the Himalayan hill region from northern Pakistan to eastern Tibet, and in Islek, Europe [1]. The common buckwheat is regularly consumed as raw or roasted groats, or as breakfast cereals, in various bakery products, and enriched non-bakery products (tea, honey, tarhana, sprouts) [2]. Because buckwheat does not contain gluten, it can be consumed by people with celiac disease [3]. The consumption of buckwheat-based products is related to a wide range of biological and healthy activities, such as hypocholesterolemic, hypoglycemic, anticancer, and anti-inflammatory, and buckwheat proteins and antioxidant phenolic compounds, such as phenolics, are presumed to be responsible, at least in part, for these benefits [3,4,5].

A new trend of cereal processing is natural and inoculated fermentation offering a wide range of derived fermented products. The fermentation processes, depending on the water content in the system, can be divided into solid- (SSF) and liquid-state fermentation (LSF). The positive aspects of cereal fermentation include the degradation of antinutrients but also increasing the nutritional value and availability of minerals, proteins, or carbohydrates [5,6,7]. Fermentation of cereals or pseudocereals is carried out mainly by lactic acid bacteria (LAB). An improvement in sensory and baking qualities was demonstrated as a result of the use of sourdough, which, through LAB metabolism, allowed us to obtain a product with an attractive flavor and texture [8]. Extended shelf life or new ingredients formed during the fermentation process are beneficial features of fermented products. However, despite these benefits, there are few reports of the effects of fermentation on plant secondary metabolites and related antioxidant properties [9]. We showed that LSF caused a slight specific LAB-dependent increase in total phenolic compounds, thus, providing evidence for the suitability of selected LAB strains to be used as natural sour agents for further bakery product development [10].

The previous study showed the average levels of phenolic acids and flavonoids in unfermented buckwheat flour, fermented flours, and water biscuits before and after in vitro digestion [11]; however, the bioaccessibility of the identified phenolic acids and flavonoids from BB_F_, despite the rutin and quercetin described bioaccessibility [12], was not investigated in relation to the specific LAB strain used for LSF. Bioaccessibility could be defined as the fraction of a compound that is released from the food matrix in the gastrointestinal lumen and used for intestinal absorption [13]. From a nutrition perspective, the measurement of bioaccessibility provides valuable information for selecting the source of food matrices to ensure the nutritional efficacy of food products [14].

Recently, we studied the multifunctionality of buckwheat biscuits (BB_F_) baked from common buckwheat flours after liquid-state fermentation (LSF) by select lactic acid bacteria (LAB). The high bioaccessible anti-AGEs activity was found after digestion in vitro of BB_F,_ which was positively correlated with the total phenolic compound bioaccessibility [15]. Moreover, we showed a low level of the ACE inhibitory activity of BB_F_ and BB_C,_ which was significantly increased after digestion. High significant correlations were found between inhibition of ACE (IC_50_) and total phenolic compounds of BB_F_ before and after digestion, thus, indicating a link between phenolic compound content and ACE inhibitory activity [11].

Therefore, this study aimed to investigate the potential bioaccessibility of individual phenolic acids and flavonoids from BB_F_ prepared from flours fermented by selected lactic acid bacteria (*L*. *acidophilus* (145, La5, V)*, L*. *casei* (*LcY, 2K)*, *L*. *delbruecki* subsp. *bulgaricus* (151, K)*, L. plantarum* (W42, IB), *L. rhamnosus* (GG, 8/4, K), *L. salivarius* AWH, *Streptococcus thermophilus* Mk-10) after an in vitro digestion procedure that mimics the physiochemical changes occurring in gastric and small intestinal digestion. 

## 2. Results

### 2.1. Bioaccessibility of Phenolic Acids

In this study, the content of the phenolic acids identified in BB_F_ and BB_C_ buckwheat biscuits before and after in vitro digestion was provided. The eight phenolic acids known as derivatives of hydroxycinnamic acid (ferulic, caffeic, sinapic, *p*-coumaric, *t*-cinnamic) and derivatives of hydroxybenzoic acid (protocatechuic, vanillic, syringic) were identified. Among phenolic acids, vanillic, protocatechuic, and syringic acids were predominant. The level of phenolic acids (μg/g DM) in BB before and after digestion in vitro is presented in Table 1 and Table 2, respectively.

Having the content of phenolic acids (μg/g DM) in buckwheat biscuits prepared from unfermented (BB_C_) and fermented flours (BB_F_) by selected lactic acid bacteria before and after in vitro digestion, the bioaccessibility indexes (BI) of phenolic acids were calculated, and they are shown in Table 3.

The content of vanillic acid in BB_F_ prepared from fermented flours ranged from 75 to 129 μg/g DM compared with 112 μg/g DM noted in the control BBc (Table 1). Digestion of biscuits led to an increase in the content of vanillic acid, and it was almost two-threefold higher for both BB_F_ and BB_C_ (Table 2). The bioaccessibility index (BI_vanillic_) for both BB_F_ and BB_C_ was >1, indicating high bioaccessibility of vanillic acid. BI_vanillic_ ranged from 1.77 to 3.22 compared with the 1.67 obtained for BBc. The highest BI_vanillic_ was found for BB_F_ baked from flour fermented by *L. plantarum* W42 and *L. rhamnosus* K (Table 3).

The protocatechuic acid was found in BB_F_ within the range of 39–85 μg/g DM compared to 65 μg/g DM noted in BBc (Table 1). After digestion of BB_F_, its content increased 4–7 times, whereas a threefold higher content was noted in digested BBc (Table 2). The bioaccessibility index (BI_protocatechuic_) for BB_F_ was >3, thus, indicating for very high bioaccessibility of this acid. BI_protocatechuic_ ranged from 2.90 to 7.79 compared with 3.09 obtained for BB_C_. The highest BI_protocatechuic_ was found for BB_F_ formulated on fermented flours by *L. rhamnosus* 8/4 and *L. salivarius* AWH (Table 3).

The syringic acid was present in BB_C_ and BB_F_ at a concentration at least threefold lower than the most abundant vanillic acid. It ranged widely from 21 to 159 μg/g DM compared with 43 μg/g DM noted in BB_C_ (Table 1). After digestion of BB_F_, its content increased significantly (Table 2), resulting in high BI_syringic_ ranging from 2.07 to 10.83 compared with 2.99 obtained for BB_C_.

The not predominant phenolic acids included *para*-coumaric, sinapic, *trans*-cinnamic, caffeic, and ferulic acid, and the following observations were drawn on the basis of their content in BB_F_ and BB_C_ before digestion (Table 1). The content of these acids in BB_C_ was from 3.4 μg/g DM (caffeic acid) up to 21.5 μg/g DM (*para*-coumaric) compared with the lowest content of 1.4 μg/g DM noted for caffeic acid in BB_F_ by *L. rhamnosus* 8/4, and the highest one of 28.5 μg/g DM found for *para*-coumaric acid in BB_F_ by *L. rhamnosus* K. Generally, the content of these acids noted in BB_F_ was decreased or not changed. There were noted some exceptions made to the selected LAB strain used for flour fermentation, where a significant increase was noted for *para*-coumaric in BB_F_ by *L. rhamnosus* K, for sinapic acid by *L. casei* 2K, for *trans*-cinnamic by *L. plantarum* W42, and for caffeic and ferulic acid in BB_F_ by *L. casei* 2K. Since the baking conditions were the same for BB_C_ and BB_F_, it is indicated for the impact of the selected LAB on the phenolic acid contents. When the total content of phenolic acids was considered, the flour fermented by *L. rhamnosus* K offered the highest content in BB_F_, higher by almost 59% compared with their content in BB_C_. 

In this study, it was found that the digestion of BB_F_ and BB_C_ led to an increase in the content of *p*-coumaric acid compared with undigested biscuits (Table 2). BI*_p_*_-coumaric_ for BB_F_ ranged from 1.06 to 5.66 compared with 1.22 noted for BB_C_ (Table 3). The highest BI*_p_*_-coumaric_ was found for BB_F_ formulated on fermented flours by *L. rhamnosus* 8/4. Similar findings were found for BI_sinapic_, BI*_t_*_-cinnamic_, and BI_ferulic,_ with the highest BI for BB_F_ formulated on fermented flours by *L. plantarum* W42 (21.79), *Streptococcus thermophilus* MK-10 (9.15), and by *L. rhamnosus* GG (4.57), respectively (Table 3). The widest range of BI was noted for caffeic acid in BB_F_ as it ranged from 3.80 up to 31.32 for fermented flours by *L. rhamnosus* 8/4. In summary, the digestion in vitro released all phenolic acids in BB_F,_ as is well seen when the average BI is compared to their BI for BB_C_ (Table 3).

### 2.2. Bioaccessibility of Flavonoids

In this study, despite rutin- (quercetin-3-rutinoside) and quercetin-described bioaccessibility in our previous study [12], seven other flavonoids before and after digestion in vitro were identified in BB_F_ and BB_C,_ including epicatechin, vitexin, orientin, apigenin, kaempferol, and luteolin (Table 4 and Table 5, respectively).

The bioaccessibility of flavonoids provided in detail (Table 6) is based on their content in BB_F_ and BB_C_ before and after digestion in vitro.

The epicatechin was the major flavonoid found in BB_F_ in a wide range from 17.9 to 127.6 μg/g DM, depending on the LAB strain used for flour fermentation compared with 91.7 μg/g DM noted for BB_C_ (Table 4). It was also found that BB_F_ contained about threefold lower epicatechin content than BB_C,_ with the exception of biscuits baked from fermented flours by *L. rhamnosus* 8/4, *L. rhamnosus* K, and *L. salivarius* AWH. The differential behavior of epicatechin was noted after digestion of BB_F,_ as in some cases, epicatechin was released from BB_F,_ or no changes were observed. However, the epicatechin level in BB_F_ after digestion was increased compared with its level in BB_C_ (Table 5). Therefore, the average BI_epicatechin_ for BB_F_ was 1.04, and it was almost five times higher compared with its value for BB_C_. The highest BI_epicatechin_ was noted for BB_F_ prepared from flours fermented by *L. acidophilus* V and *L. acidophilus* 145 (Table 6).

A similar trend was noted for vitexin, orientin, apigenin, and luteolin; however, their content in BB_F_ was lower than epicatechin. Their BI indexes were higher than one, indicating the high bioaccessibility in contrast to the BI value lower than one noted for BB_C_. The opposite data were provided for kaempferol since its content was decreased after digestion of both BB_F_ and BB_C_ (Table 5), but its bioaccessibility was still very high (Table 6).

**Figure 1:**
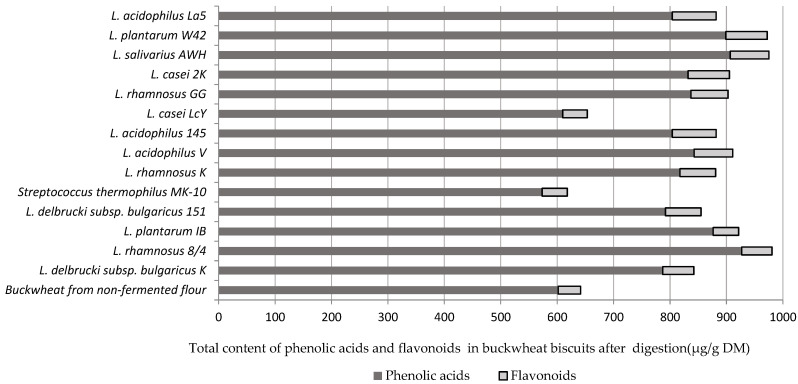
The summed-up content of phenolic acids and flavonoids in buckwheat biscuits prepared from fermented flours by selected lactic acid bacteria.

As shown in Figure 1, all LAB strains used for buckwheat flour fermentation, despite *L. casei* LcY and *Streptococus thermophilus* MK-10, offered a buckwheat dough matrice from which phenolic acids and flavonoids were easier released into digestion fluid. It was noted that phenolic acids formed the main fraction after digestion in vitro compared with flavonoids.

## 3. Discussion

### 3.1. Bioaccessibility of Phenolic Acids

There is an increasing interest in a healthy lifestyle and the consumption of substantial portions of secondary plant metabolites, such as polyphenols, because of their benefits for the human body. As human studies are time-consuming, costly, and restricted by ethical concerns, in vitro models for investigating the effects of digestion on these compounds have been developed to predict their release from the food matrix, as well as their bioaccessibility [16]. The most widely used procedure for screening polyphenolic compound bioaccessibility is the in vitro static GI method [17]. Contrary evidence on the bioaccessibility of phenolic compounds is available in the literature. Carbonell-Capella et al. [17] showed that gastric digestion increased polyphenolic concentration, whereas the duodenal fraction significantly diminished polyphenolic content. In contrast, Tagliazucchi et al. [18] observed an increase in the bioaccessibility of total polyphenols, flavonoids, and anthocyanins during the gastric digestion in grapes, while intestinal digestion caused a decrease in all classes of polyphenols.

It was shown that in vitro digestion released much higher levels of total phenolic compounds (TPC) from biscuits obtained from fermented buckwheat flour compared with biscuits before digestion, which indicated a much better extraction system for phenolic compounds, which was the digestion fluid, compared with the classical extraction [11]. Generally, an increase in the potential bioaccessibility of TPC was observed. As a consequence, the individual phenolic compounds responsible for this increase in the bioaccessibility of TPC should be indicated. The data on the bioaccessibility of phenolic acids from the buckwheat matrix modified by the use of fermented flour for baking are still limited. In this study, it was shown that vanillic, protocatechuic, and syringic acids were predominant in buckwheat biscuits (control and obtained from fermented flour). Previously it was shown that the baking of BB_F_ and BB_C_ resulted in a reduction in the average content of phenolic acids [11]. Heat treatment may enhance polyphenol bioaccessibility because of disruption of plant tissue and denaturation of polyphenols–polysaccharide complexes. However, heat treatment may also cause thermal degradation of phenolic compounds [19]. As was presented in the review by Wojtunik-Kulesza et al. [20] during consideration of in vitro bioaccessibility studies, chemical and biochemical reactions or physical constraints occurring within food must be taken into account. Additionally, the release from the food matrix, particle size or pH-dependent transformations, and interactions between polyphenols and food components should be taken into account. For example, it was shown that the bioaccessibility of sinapic acid from bran-rich bread was much higher than that of ferulic acid and *para*-coumaric acid [21]. However, most phenolic compounds remain stable during salivary and gastric digestion [22]. Managa et al. [23] demonstrated that lactic acid bacteria used for fermentation of a smoothie composed of pineapple and chayote leaves increase the total phenol. These authors found that after in vitro digestion, fermentation improved the total phenol recovery by 66% during the intestinal phase compared with the control sample. After digestion, the TPC of mango juices decreased, while LAB-fermentation improved its bioaccessibility [24]. Bloem et al. [25] showed that *Oenococcus oeni* was not able to convert vanillic acid into vanillin. Micro-organisms, such as yeast, are also able to metabolize vanillin to vanillic acid or vanillyl alcohol by oxidoreductase enzymes [26]. Phelps and Young [27] demonstrated that the plant phenolic compounds ferulic and syringic acid were readily degraded by consortia of bacteria from this site under methanogenic, sulfidogenic, and denitrifying conditions.

### 3.2. Bioaccessibility of Flavonoids

Since the beneficial health effects of flavonoids depend on their absorption in the gut [28,29], their bioaccessibility is important to indicate their possible influence on the human organism. Rutin is the main buckwheat flavonoid, whereas quercetin is present in significantly lower concentrations [30], and our previous investigation showed that fermentation, baking, and in vitro digestion significantly affect their content [12]. It was found that the expanded bioaccessibility of rutin from BB_F_ was low, and the BI of quercetin was greater than 1. Payne et al. [31] found that epicatechin, compared with catechin, is as much as 30 times greater in fresh and dried cocoa beans, but as conventional processing occurs, there is a loss in epicatechin and, at times, an increase in catechin.

Choi et al. [32] showed that the total flavonoid contents of the various buckwheat food matrices were higher after digestion compared with the predigested form, which indicated that flavonoids are easily released by in vitro digestion. These authors found that processed buckwheat samples had improved flavonoid bioaccessibility upon baking, which indicated that they are easily released from the food matrix by both digestion and baking. A significant increase of 7 out of 11 flavonoid compounds after in vitro gastrointestinal digestion of quinoa products was presented by Balakrishnan and Schneider [33]. Thilakarathna and Rupasinghe [34], in the review, showed that flavonoids had shown promising health-promoting effects in human cell culture, experimental animal, and human clinical studies. Still, an investigation is required to enhance the bioavailability and subsequent efficacy of certain flavonoids using consumer-friendly technologies.

## 4. Materials and Methods

### 4.1. Chemicals

Reagents in MS grade, including acetonitrile, methanol, water, and formic acid, were purchased from Sigma Chemical Co. (St. Louis, MO, USA). While diethyl ether (Et2O), hydrochloric acid (HCl), and sodium hydroxide (NaOH) were obtained from POCH S.A. (Gliwice, Poland). Compound standards (phenolic acids, flavonoids) were purchased from Sigma Chemical Co. (St. Louis, MO, USA) and Extrasynthese (Genay, France) and were used for identification and calculation.

### 4.2. Fermentation of Buckwheat Flours by LAB, Preparation of Buckwheat Biscuits from Fermented Flours (BB_F_), and In Vitro Digestion of BB_F_

#### 4.2.1. Buckwheat Flour

Buckwheat flour originating from commercial Polish common buckwheat (Fagopyrum esculentum Moench) was purchased from local industry (Melvit S.A., Kruki, Poland). According to the produced declaration, the carbohydrate, dietary fiber, proteins, and fat content of buckwheat flour and roasted buckwheat groats were 62%, 2.3%, 7.2%, and 0.7% on a dry basis, respectively. Before fermentation, the buckwheat flour was pretreated as follows: about 50 g of flour was suspended with 950 mL of distilled water, heated at 90 °C for 45 min, then autoclaved at 121 °C for 15 min, and finally cooled to 37 °C. The pretreatment was carried out to reduce microbial populations in buckwheat flour before fermentation since they would compete with and inhibit the growth of inoculated microbes during the fermentation process.

#### 4.2.2. Fermentation of Buckwheat Flours

The following selected lactic acid bacteria were used: *L. acidophilus* (145, La5, V); *L. casei* (LcY, 2K); *L. delbruecki* subsp. *bulgaricus* (151, K); *L. plantarum* (W42, IB); *L. rhamnosus* (GG, 8/4, K); *L. salivarius* AWH and *Strepcococcus thermophilus* Mk-10, all strains originated from the Institute of Animal Reproduction and Food Research of Polish Academy of Sciences’ collections. The *Lactobacillus rhamnosus* GG was purchased from ATCC^®^. Fermentation of buckwheat flours was carried out as follows: the 5% suspension of pretreated buckwheat flour in distilled water was inoculated with selected lactic acid bacteria with an amount of 8.00 log CFU/mL, and fermentation was performed at 37 °C for 24 h. The pretreated buckwheat flour not subjected to a fermentation process was used as a control sample. After fermentation, the samples were freeze-dried (Christ—Epsilon 2-6D LSC plus, Germany).

#### 4.2.3. Preparation of BB_F_ from Fermented Flour

The biscuit dough was prepared according to the AACC 10–52 method [35], with the modification proposed by Hidalgo and Brandolini [36]. The dough was cut with a square cookie cutter (60 mm). BBs were baked at 220 °C for 30 min (electric oven DC-21 model, Sveba Dahlen AB, Fristad, Sweden). The control biscuits (BBc) were formulated on unfermented buckwheat flour. The buckwheat biscuits were lyophilized, milled, and stored in a refrigerator until analysis.

#### 4.2.4. In Vitro Digestion of Buckwheat Biscuits

The BB_F_ and BB_C_ were in vitro digested as described by Delgado-Andrade et al. [37] with some modifications [38]. Briefly, the three steps of digestion were saliva (pH 7.0), gastric (pH 2.0), and intestinal digestion (pH 7.5). Briefly, 10 g of lyophilized and milled buckwheat biscuits was suspended in 80 mL of deionized water. An α-amylase solution (77 U/mg solid) was added to the samples at a proportion of 3.25 mg/10 g of sample dry matter (d.m.) in 1 mM CaCl_2_, pH 7.0. Then, samples were shaken in a water bath at 37 °C for 30 min. For gastric digestion, the pH was reduced to 2.0 with 6 N HCl, and pepsin solution (738 U/mg) was added in the amount of 0.5 g/10 g of sample d.m. in 0.1 N HCl. The incubation was continued under the same conditions for 120 min. In the next step, the pH was adjusted to 6.0 with 6 M NaOH, and a mixture of pancreatin (activity 8xUSP) and bile salts extract was added. Subsequently, the pH was increased to 7.5 with 6 M NaOH, and water buffering to a pH of 7.5 was introduced to obtain a final volume of 150 mL. Then, the samples were incubated at 37 °C for 120 min. After incubation, the digestive enzymes were inactivated by heating at 100 °C for 4 min and cooled for centrifugation at 5000 rpm for 60 min at 4 °C in an MPV-350R centrifuge (MPW Med. Instruments, Warsaw, Poland). The supernatants obtained were stored at −18 °C for the evaluation of the bioaccessibility of phenolic acids and flavonoids from water biscuits.

### 4.3. Extraction, Isolation, and HPLC Analysis of Phenolic Compounds from BB_F_ before and after In Vitro Digestion

The analysis of polyphenols (phenolic acids and flavonoids) was conducted according to the modified method of Wiczkowski et al. [39]. In the first step, about 0.05 g of freeze-dried samples was extracted 5 times with 80% MeOH. Next, polyphenolic compounds (forms released from soluble esters and soluble glycosides as well as free forms) were separated from the methanolic extracts in several stages. In the case of free forms of polyphenols, after adjusting the primary extract to pH 2 with 6 M HCl, the isolation by diethyl ether was carried out. However, in the case of conjugated forms (esters and glycosides), before adjusting the extract to pH 2 and the extraction of released forms of polyphenols by diethyl ether, the hydrolysis under a nitrogen atmosphere was executed for 4 h at room temperature with 4 M NaOH and subsequently in the condition of 6 M HCl for 1 h at 100 °C. After each hydrolysis, the extraction process was conducted in triplicates by utilizing sonication and centrifugation, and the collected ether extracts were evaporated to dryness under a nitrogen atmosphere at 35 °C. For the analysis of the profile and content of phenolic acids and flavonoids, the HPLC system (LC-200, Eksigent, Vaughan, ON, Canada) coupled with a mass spectrometer (QTRAP 5500, AB Sciex, Vaughan, ON, Canada) consisting of a triple quadrupole, ion trap, and ion source of electrospray ionization (ESI) was used. The chromatographic separation was conducted with a HALO C_18_ column (50 mm × 0.5 mm × 2.7 μm, Eksigent, Vaughan, ON, Canada) at 45 °C, at the flow rate of 15 μL/min. Identification and quantitation of the phenolic acids and flavonoids were based on the comparison of their retention times and the presence of the respective parent and daughter ion pairs (Multiple Reaction Monitoring method, MRM) with data obtained after analysis of the authentic standards.

### 4.4. Calculation of the Bioaccessibility Index of Phenolic Compounds

In this study, we determined the bioaccessibility index (BI) [38] of individual phenolic acids and flavonoids, which was calculated according to the following formulas:

BI_PA_ = PA_GD_/FA_BB_ and BI_F_ = F_GD_/F_BB_

where PA_GD_ is the indicated phenolic acid content after simulated gastrointestinal digestion (GD), F_GD_ is indicated flavonoid content after simulated gastrointestinal digestion (GD), PA_BB_ is the indicated phenolic acid content in BB, and F_BB_ is indicated flavonoid content in BB. BI_PA_ and BI_F_ values > 1 indicate high bioaccessibility of phenolic acids and flavonoids from BB (BB_F_ and BB_C)_; BI_FA_ and BI_F_ values < 1 indicate low bioaccessibility.

### 4.5. Statistical Analysis

Results are given as the average ± standard deviation (SD) of *n* = 3 independent experiments. They were determined by one-way analysis of variance (ANOVA) with Fisher’s least significant difference test (*p* < 0.05). All analyses were made using STATISTICA for Windows (StatSoft Inc., Tulsa, OK, USA, 2001).

## 5. Conclusions

The bioaccessibility indexes of phenolic acids and flavonoids from buckwheat biscuits formulated from flours fermented by selected LAB are important factors in understanding the bioavailability of these compounds. The eight phenolic acids (protocatechuic, vanillic, syringic, ferulic, caffeic, sinapic, *p*-coumaric, and *t*-cinnamic) and seven other flavonoids than rutin and quercetin, including epicatechin, vitexin, orientin, apigenin, kaempferol, and luteolin were identified in buckwheat biscuits before and after digestion in vitro. The obtained data indicated the high bioaccessibility of phenolic acids and improved bioaccessibility of flavonoids under the influence of the fermentation and baking processes used. The study provides evidence for the suitability of selected LAB strains to be used as natural selected sour agents for further bakery product development rich in indicated phenolic acids and flavonoids with high bioaccessibility.

## Figures and Tables

**Table 1:** The content of phenolic acids (μg/g DM) in buckwheat biscuits prepared from unfermented (BB_C_) and fermented flours (BB_F_) by selected lactic acid bacteria. Data are expressed as mean ± standard deviation (*n* = 3). Means in each column followed by different letters are significantly different (*p* < 0.05) based on the one-way analysis of variance (ANOVA).

Sample/Phenolic Acid	Vanillic	Protocatechuic	Syringic	*p*-Coumaric	Sinapic	*t*-Cinnamic	Caffeic	Ferulic
*Control biscuits (BBc)*	112.66 ± 2.66b	65.79 ± 2.46bc	43.63 ± 1.33d	21.53 ± 3.10b	8.33 ± 0.10c	7.86 ± 0.02d	3.40 ± 0.11c	3.21 ± 0.13cd
*BB_F_ fermented by:*								
*L. plantarum* IB	84.41 ± 3.29c	73.48 ± 2.40b	53.83 ± 3.03c	16.29 ± 0.76c	2.47 ± 0.07f	13.15 ± 0.62ab	3.54 ± 0.08c	3.76 ± 0.13c
*L. plantarum* W42	75.60 ± 2.49d	85.48 ± 1.96a	50.44 ± 2.18c	13.50 ± 3.64cd	1.29 ± 0.05g	14.07 ± 0.49a	2.27 ± 0.14cd	3.64 ± 0.11c
*L. delbrucki* subsp. *bulgaricus* 151	97.14 ± 6.39c	52.71 ± 1.97c	34.71 ± 0.46d	7.58 ± 0.36e	5.57 ± 0.24d	2.82 ± 0.14e	4.21 ± 0.07c	2.47 ± 0.09e
*L. casei* Lcy	95.40 ± 3.37c	78.82 ± 0.98a	36.93 ± 0.19d	6.37 ± 0.08e	4.76 ± 0.15e	11.33 ± 1.01b	3.95 ± 0.05c	2.71 ± 0.11e
*Streptococcus thermophilus* MK-10	111.15 ± 4.17b	39.72 ± 2.07d	38.48 ± 0.66d	8.26 ± 0.54e	6.67 ± 0.15d	1.87 ± 0.05e	4.45 ± 0.11c	2.51 ± 0.07e
*L. acidophilus* La5	108.18 ± 2.26b	81.98 ± 4.05a	46.56 ± 0.81c	11.33 ± 0.21c	7.77 ± 0.29cd	3.05 ± 0.17e	11.68 ± 0.73a	4.29 ± 0.10b
*L. acidophilus* V	115.33 ± 3.10b	82.47 ± 1.00a	60.81 ± 2.19bc	9.67 ± 0.24d	8.24 ± 0.15c	4.35 ± 0.09e	11.93 ± 1.11a	4.17 ± 0.14bc
*L. acidophilus* 145	104.28 ± 0.37b	61.19 ± 4.57c	52.36 ± 0.55bc	4.00 ± 0.12e	6.20 ± 0.13d	11.65 ± 0.50b	2.49 ± 0.07cd	3.23 ± 0.04c
*L. casei* 2K	112.01 ± 1.56b	57.00 ± 1.51c	36.94 ± 1.53d	12.72 ± 0.56cd	22.35 ± 0.34a	3.09 ± 0.02e	10.30 ± 0.34a	6.00 ± 0.10a
*L. delbrucki* subsp. *bulgaricus* K	83.06 ± 1.59cd	42.57 ± 0.30d	21.42 ± 0.49e	8.30 ± 0.35d	4.35 ± 0.12e	3.09 ± 0.03e	1.59 ± 0.05d	2.97 ± 0.11d
*L. rhamnosus* GG	100.91 ± 0.84b	52.52 ± 2.62c	35.64 ± 0.96d	13.68 ± 0.16cd	16.24 ± 0.35b	5.04 ± 0.35e	7.94 ± 0.36b	2.41 ± 0.06e
*L. rhamnosus* 8/4	97.22 ± 2.22c	43.59 ± 0.50d	45.50 ± 1.85cd	3.41 ± 0.02e	4.20 ± 0.04e	5.27 ± 0.11e	1.36 ± 0.04d	3.44 ± 0.05c
*L. rhamnosus* K	129.02 ± 2.65a	73.90 ± 3.60b	159.66 ± 6.62a	28.53 ± 0.64a	7.12 ± 0.38d	10.00 ± 0.53c	11.68 ± 0.35a	3.19 ± 0.14cd
*L. salivarius* AWH	95.95 ± 6.11c	50.33 ± 1.71c	35.30 ± 1.77d	7.64 ± 0.32de	8.64 ± 0.78c	6.12 ± 0.28e	3.17 ± 0.12c	2.90 ± 0.12d
*Average for BB_F_*	100.69 ± 14.15	62.55 ± 16.34	50.61 ± 32.98	10.81 ± 6.32	7.56 ± 5.53	6.78 ± 4.31	5.75 ± 4.04	3.41 ± 0.96

**Table 2:** The content of phenolic acids (μg/g DM) in buckwheat biscuits prepared from not fermented (BB_C_) and fermented flours (BB_F_) by selected lactic acid bacteria after in vitro digestion. Data are expressed as mean ± standard deviation (*n* = 3). Means in each column followed by different letters are significantly different (*p* < 0.05) based on the one-way analysis of variance (ANOVA).

Sample/Phenolic Acid	Vanillic	Protocatechuic	Syringic	*p*-Coumaric	Sinapic	*t*-Cinnamic	Caffeic	Ferulic
*Control biscuits (BBc)*	187.90 ± 18.83c	203.57 ± 6.15d	130.65 ± 1.22c	26.33 ± 0.14e	22.16 ± 0.30b	8.29 ± 0.02f	14.64 ± 0.09e	8.17 ± 0.21c
*BB_F_ fermented by:*								
*L. plantarum* IB	197.89 ± 6.37c	327.77 ± 3.64a	208.15 ± 6.37ab	23.60 ± 0.19f	24.22 ± 0.86b	46.90 ± 0.26a	37.38 ± 0.52c	10.48 ± 0.18b
*L. plantarum* W42	243.73 ± 5.25b	297.80 ± 7.81b	199.22 ± 4.88ab	26.36 ± 0.30e	28.11 ± 0.74ab	40.53 ± 0.95b	52.54 ± 0.76a	10.61 ± 0.16b
*L. delbrucki* subsp. *bulgaricus* 151	212.09 ± 5.68c	242.62 ± 4.62d	211.32 ± 1.23a	28.75 ± 0.26e	29.93 ± 0.60a	12.41 ± 0.34f	44.38 ± 0.48b	10.38 ± 0.25b
*L. casei* Lcy	226.05 ± 1.41c	228.62 ± 2.12d	76.55 ± 2.21d	14.14 ± 0.24h	6.77 ± 0.05e	29.31 ± 0.91d	21.98 ± 0.15d	6.37 ± 0.09c
*Streptococcus thermophilus* MK-10	236.60 ± 2.85b	166.90 ± 3.99d	85.61 ± 1.61d	19.59 ± 0.44g	13.39 ± 0.26d	17.10 ± 0.72e	27.18 ± 0.61d	6.95 ± 0.04c
*L. acidophilus* La5	233.95 ± 4.89b	271.34 ± 9.51c	184.67 ± 4.79b	47.08 ± 1.25a	32.12 ± 0.27a	15.24 ± 0.17e	57.05 ± 0.27a	11.94 ± 0.14a
*L. acidophilus* V	244.10 ± 5.66b	285.54 ± 1.17b	174.31 ± 4.23b	40.18 ± 0.85b	25.30 ± 1.04b	17.51 ± 0.53e	45.29 ± 1.35b	10.44 ± 0.17b
*L. acidophilus* 145	247.57 ± 8.06b	258.21 ± 6.99c	198.11 ± 8.79ab	17.01 ± 0.24g	15.07 ± 0.36cd	34.73 ± 0.21c	23.50 ± 0.19d	9.71 ± 0.15b
*L. casei* 2K	233.78 ± 6.70b	291.43 ± 3.88b	154.45 ± 3.99c	41.87 ± 0.45b	30.52 ± 1.58a	17.57 ± 0.4e	51.27 ± 1.43a	11.14 ± 0.29b
*L. delbrucki* subsp. *bulgaricus* K	233.54 ± 1.97b	210.31 ± 3.64d	231.97 ± 3.90a	27.14 ± 0.28e	25.62 ± 0.54b	11.59 ± 0.20f	36.72 ± 1.31c	10.26 ± 0.28b
*L. rhamnosus* GG	268.28 ± 14.23a	245.80 ± 6.36c	177.38 ± 7.54b	36.67 ± 0.47c	29.11 ± 0.13a	19.80 ± 0.67d	49.08 ± 0.27ab	11.01 ± 0.25b
*L. rhamnosus* 8/4	266.25 ± 14.04a	339.72 ± 8.67a	212.00 ± 8.22a	19.29 ± 0.44g	17.34 ± 0.08c	19.58 ± 0.83d	42.53 ± 0.60c	10.43 ± 0.10b
*L. rhamnosus* K	228.37 ± 1.05c	265.03 ± 3.65b	186.60 ± 6.75ab	30.15 ± 0.29de	30.09 ± 0.78a	14.49 ± 0.10e	52.30 ± 1.08a	10.49 ± 0.18b
*L. salivarius* AWH	281.37 ± 10.60a	307.99 ± 2.40ab	180.70 ± 4.93ab	30.33 ± 0.47de	31.22 ± 0.93a	18.89 ± 0.49d	46.38 ± 1.47b	9.88 ± 0.22b
*Average for BB_F_*	239.54 ± 22.01	267.08 ± 46.74	177.22 ± 45.11	28.73 ± 9.87	24.20 ± 7.91	22.55 ± 10.96	41.97 ± 11.19	10.01 ± 1.52

**Table 3:** The bioaccessibility indexes (BI) of phenolic acids from buckwheat biscuits prepared from unfermented (BB_C_) and fermented flours (BB_F_) by selected lactic acid bacteria.

Sample/Phenolic Acid	Vanillic	Protocatechuic	Syringic	*p*-Coumaric	Sinapic	*t*-Cinnamic	Caffeic	Ferulic
*Control biscuits (BB_C_)*	1.67	3.09	2.99	1.22	2.66	1.05	4.31	2.55
*BB_F_ fermented by:*								
*L. plantarum* IB	2.34	4.46	3.87	1.45	9.81	3.57	10.56	2.79
*L. plantarum* W42	3.22	3.48	3.95	1.95	21.79	2.88	23.18	2.92
*L. delbrucki* subsp. *bulgaricus* 151	2.18	4.60	6.09	3.79	5.37	4.40	10.55	4.20
*L. casei* Lcy	2.37	2.90	2.07	2.22	1.42	2.59	5.57	2.35
*Streptococcus thermophilus* MK-10	2.13	4.20	2.22	2.37	2.01	9.15	6.11	2.77
*L. acidophilus* La5	2.16	3.31	3.97	4.15	4.13	4.99	4.88	2.78
*L. acidophilus* V	2.12	3.46	2.87	4.15	3.07	4.03	3.80	2.50
*L. acidophilus* 145	2.37	4.22	3.78	4.25	2.43	2.98	9.44	3.01
*L. casei* 2K	2.09	5.11	4.18	3.29	1.37	5.68	4.98	1.86
*L. delbrucki* subsp. *bulgaricus* K	2.81	4.94	10.83	3.27	5.89	3.76	23.12	3.45
*L. rhamnosus* GG	2.66	4.68	4.98	2.68	1.79	3.93	6.18	4.57
*L. rhamnosus* 8/4	2.74	7.79	4.66	5.66	4.13	3.71	31.32	3.03
*L. rhamnosus* K	1.77	3.59	1.17	1.06	4.23	1.45	4.48	3.29
*L. salivarius* AWH	2.93	6.12	5.12	3.97	3.61	3.09	14.63	3.41
*Average for BB_F_*	2.4 ± 0.4	4.5 ± 1.3	4.3 ± 2.3	3.2 ± 1.3	5.1 ± 5.3	4.0 ± 1.8	11.3 ± 8.6	3.1 ± 0.7

**Table 4:** The content of flavonoids (μg/g DM) in buckwheat biscuits prepared from not fermented (BBc) and fermented flours (BB_F_) by selected lactic acid bacteria. Data are expressed as mean ± standard deviation (*n* = 3). Means in each column followed by different letters are significantly different (*p* < 0.05) based on the one-way analysis of variance (ANOVA).

Strain/Flavonoid	Epicatechin	Vitexin	Orientin	Apigenin	Kaempferol	Luteolin
*Control biscuits (BBc)*	91.69 ± 2.73c	15.04 ± 0.21b	4.21 ± 0.18b	2.13 ± 0.20c	0.75 ± 0.12b	0.22 ± 0.02ab
*BB_F_ fermented by:*						
*L. plantarum* IB	19.13 ± 0.33e	7.81 ± 0.15d	2.24 ± 0.04d	2.91 ± 0.14b	0.68 ± 0.05b	0.17 ± 0.01ab
*L. plantarum* W42	32.50 ± 1.48d	14.00 ± 0.11b	2.53 ± 0.02d	2.46 ± 0.06c	0.89 ± 0.02b	0.14 ± 0.01b
*L. delbrucki* subsp. *bulgaricus* 151	42.77 ± 2.15d	14.93 ± 0.26b	4.40 ± 0.20b	3.41 ± 0.18a	0.82 ± 0.07b	0.13 ± 0.01b
*L. casei* Lcy	41.57 ± 1.35d	12.74 ± 0.18c	3.10 ± 0.05cd	2.30 ± 0.09c	0.73 ± 0.04b	0.12 ± 0.03b
*Streptococcus thermophilus* MK-10	60.54 ± 1.90d	10.09 ± 0.23d	3.13 ± 0.09c	2.39 ± 0.04c	0.52 ± 0.02c	0.09 ± 0.01b
*L. acidophilus* La5	47.39 ± 4.28d	11.77 ± 0.23c	2.77 ± 0.10d	1.94 ± 0.04d	0.45 ± 0.01c	0.12 ± 0.01b
*L. acidophilus* V	23.20 ± 0.56e	11.84 ± 0.33c	3.02 ± 0.07cd	1.99 ± 0.08d	0.47 ± 0.05c	0.12 ± 0.01b
*L. acidophilus* 145	17.92 ± 0.43e	10.00 ± 0.13d	3.17 ± 0.04c	1.98 ± 0.10d	0.45 ± 0.20c	0.15 ± 0.02ab
*L. casei* 2K	40.96 ± 0.84d	11.03 ± 0.08c	2.53 ± 0.12d	2.63 ± 0.06c	0.60 ± 0.16c	0.16 ± 0.02ab
*L. delbrucki* subsp. *bulgaricus* K	20.60 ± 0.49e	9.80 ± 0.29d	4.64 ± 0.19b	0.67 ± 0.01e	0.57 ± 0.02c	0.10 ± 0.01b
*L. rhamnosus* GG	49.87 ± 2.67d	10.98 ± 0.28d	2.06 ± 0.15e	2.86 ± 0.11b	0.57 ± 0.12c	0.17 ± 0.03ab
*L. rhamnosus* 8/4	101.47 ± 6.09c	11.61 ± 0.21c	3.19 ± 0.09c	2.71 ± 0.08b	1.58 ± 0.06a	0.22 ± 0.05ab
*L. rhamnosus* K	114.57 ± 3.60b	12.27 ± 0.15c	11.48 ± 0.35a	2.56 ± 0.09c	0.97 ± 0.24b	0.25 ± 0.04a
*L. salivarius* AWH	127.64 ± 4.89a	21.96 ± 0.64a	3.62 ± 0.13c	3.02 ± 0.09a	1.07 ± 0.11b	0.15 ± 0.05ab
*Average for BB_F_*	52.87 ± 36.08	12.2 ± 3.33	3.71 ± 2.35	2.42 ± 0.66	0.74 ± 0.31	0.15 ± 0.04

**Table 5:** The content of flavonoids (μg/g DM) in buckwheat biscuits prepared from not fermented (BB_C_) and fermented flours (BB_F_) by selected lactic acid bacteria after in vitro digestion. Data are expressed as mean ± standard deviation (*n* = 3). Means in each column followed by different letters are significantly different (*p* < 0.05) based on the one-way analysis of variance (ANOVA).

Strain/Flavonoid	Epicatechin	Vitexin	Orientin	Apigenin	Kaempferol	Luteolin
*Control biscuits (BBc)*	16.45 ± 0.53d	8.30 ± 0.29d	4.23 ± 0.04d	1.25 ± 0.02e	9.27 ± 0.08a	0.19 ± 0.02b
*BB_F_ fermented by:*						
*L. plantarum* IB	22.35 ± 0.83d	12.50 ± 0.08e	6.49 ± 0.21b	1.74 ± 0.01e	1.99 ± 0.02d	0.22 ± 0.02b
*L. plantarum* W42	42.48 ± 1.45bc	17.51 ± 0.15b	8.14 ± 0.20a	2.45 ± 0.05d	2.51 ± 0.04c	0.24 ± 0.02b
*L. delbrucki* subsp. *bulgaricus* 151	37.27 ± 1.63c	14.88 ± 0.50c	6.49 ± 0.24b	2.15 ± 0.03de	2.06 ± 0.03cd	0.18 ± 0.01bc
*L. casei* Lcy	23.69 ± 1.22d	11.65 ± 0.22e	4.53 ± 0.15d	1.68 ± 0.06e	1.74 ± 0.07e	0.15 ± 0.01c
*Streptococcus thermophilus* MK-10	18.12 ± 0.98d	11.84 ± 0.10e	6.14 ± 0.04b	6.89 ± 0.07b	1.40 ± 0.02e	0.14 ± 0.03c
*L. acidophilus* La5	56.07 ± 0.91a	14.08 ± 0.18c	4.70 ± 0.02cd	11.66 ± 0.30a	1.83 ± 0.03d	0.20 ± 0.01b
*L. acidophilus* V	44.49 ± 0.89b	14.33 ± 0.30c	4.78 ± 0.06cd	2.77 ± 0.07d	1.86 ± 0.03d	0.19 ± 0.01b
*L. acidophilus* 145	49.26 ± 1.57b	17.13 ± 0.21b	6.12 ± 0.28b	2.94 ± 0.12d	2.13 ± 0.03c	0.31 ± 0.02a
*L. casei* 2K	41.34 ± 0.98b	19.01 ± 0.29a	7.50 ± 0.13a	3.15 ± 0.10d	1.93 ± 0.03d	0.30 ± 0.01a
*L. delbrucki* subsp. *bulgaricus* K	32.93 ± 0.77c	12.06 ± 0.15e	4.62 ± 0.11cd	2.26 ± 0.09e	3.06 ± 0.04b	0.15 ± 0.02bc
*L. rhamnosus* GG	42.16 ± 2.44b	12.18 ± 0.23e	6.28 ± 0.16b	2.82 ± 0.07d	2.14 ± 0.02cd	0.16 ± 0.01bc
*L. rhamnosus* 8/4	29.38 ± 0.89c	13.99 ± 0.35c	6.12 ± 0.17b	2.09 ± 0.06e	1.85 ± 0.03d	0.19 ± 0.00b
*L. rhamnosus* K	39.21 ± 1.52b	13.40 ± 0.15d	5.77 ± 0.06c	2.90 ± 0.03d	1.84 ± 0.03d	0.20 ± 0.01b
*L. salivarius* AWH	43.20 ± 2.22b	12.40 ± 0.28e	5.93 ± 0.21b	4.25 ± 0.09c	2.53 ± 0.06c	0.19 ± 0.01b
*Average for BB_F_*	37.28 ± 10.78	14.07 ± 2.24	5.97 ± 1.06	3.55 ± 2.68	2.06 ± 0.41	0.20 ± 0.05

**Table 6:** Bioaccessibility indexes (BI) of flavonoids from buckwheat biscuits prepared from unfermented (BB_C_) and fermented (BB_F_) flours by selected lactic acid bacteria.

Strain/Flavonoid	Epicatechin	Vitexin	Orientin	Apigenin	Kaempferol	Luteolin
*Control biscuits (BBc)*	0.18	0.55	1.01	0.59	12.29	0.87
*BB_F_ fermented by:*						
*L. plantarum* IB	1.16	1.60	2.90	0.60	2.95	1.27
*L. plantarum* W42	1.31	1.25	3.22	1.00	2.84	1.71
*L. delbrucki* subsp. *bulgaricus* 151	0.87	1.00	1.48	0.63	2.52	1.39
*L. casei* Lcy	0.57	0.91	1.46	0.73	2.38	1.25
*Streptococcus thermophilus* MK-10	0.30	1.17	1.96	2.88	2.68	1.63
*L. acidophilus* La5	1.18	1.20	1.69	6.01	4.03	1.74
*L. acidophilus* V	1.92	1.21	1.58	1.39	3.95	1.57
*L. acidophilus* 145	2.75	1.71	1.93	1.49	4.68	2.07
*L. casei* 2K	1.01	1.72	2.97	1.20	3.23	1.93
*L. delbrucki* subsp. *bulgaricus* K	1.60	1.23	1.00	3.37	5.34	1.48
*L. rhamnosus* GG	0.85	1.11	3.05	0.98	3.74	0.98
*L. rhamnosus* 8/4	0.29	1.21	1.92	0.77	1.17	0.86
*L. rhamnosus* K	0.34	1.09	0.50	1.13	1.89	0.79
*L. salivarius* AWH	0.34	0.56	1.64	1.41	2.37	1.28
*Average for BB_F_*	1.04	1.21	1.95	1.69	3.13	1.43

## Data Availability

Data sharing is not applicable.

## Sample Availability

Not available.
